# Supplementary material for: The conserved NxNNWHW motif in Aha-type co-chaperones modulates the kinetics of Hsp90 ATPase stimulation
Source: Nat Commun. 2019 Mar 20;10:1273. doi: 10.1038/s41467-019-09299-3 (PMC6426937; doi:10.1038/s41467-019-09299-3)
Supplement: Supplementary file 1 — Supplementary Information [file 41467_2019_9299_MOESM1_ESM.pdf]

**Supplementary information**

The conserved NxNNWHW Motif in Aha-type co-chaperones modulates the kinetics of Hsp90 ATPase stimulation

**Mercier et. al.**

# Supplementary figure 1

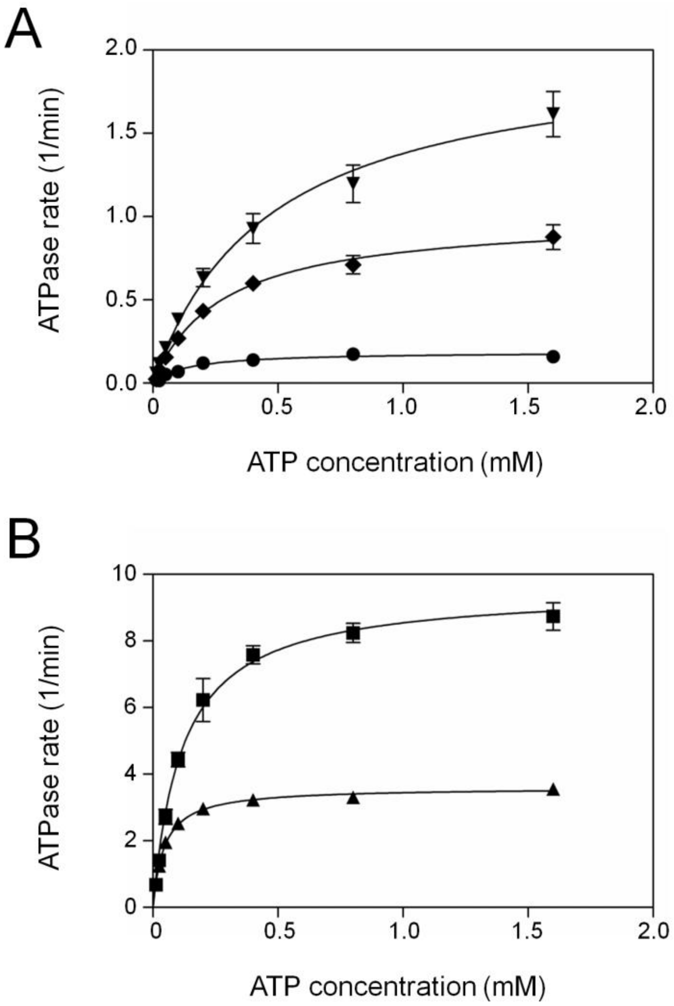


**Supplementary Figure 1**. The NxNNWHW motif in Aha1p and Hch1p modulates the apparent $K_M$ for ATP of Hsp90. A. Kinetic analysis was carried out for Hsp82p alone (circles) and in the presence of Hch1p (down triangles) or Hch1p$^{\Delta 11}$ (diamonds). B. Kinetic analysis was carried out for Hsp82p in the presence of Aha1p (squares) or Aha1p$^{\Delta 11}$ (up triangles). ATPase reactions were carried out with increasing concentrations of ATP (12.5, 25, 50, 100, 200, 400, 800, 1600 µM) and ATPase rates were analyzed with the Michaelis-Menten non-linear regression function in GraphPad Prism. The curve fits all had $R^2$ values greater than 0.9 (n=3; n=4 for Hsp82p alone). 2 µM of Hsp82p was added to reactions containing 20 µM of either Aha1p or Aha1p$^{\Delta 11}$ or 4 µM of Hsp82p was added to reactions containing 40 µM Hch1p or Hch1p$^{\Delta 11}$. 4 µM of Hsp82p was used in reactions containing Hsp82p alone.

# Supplementary figure 2

| Primer # | Primer name | Primer sequence |
|---|---|---|
| 13 | senseScAha1BamHI | gagagaggatccatggtcgtgaataacccaaataactg |
| 16 | antisenseScAha1mycXhoI | gagagactcgagtcactacaaatcttcttcagaaatcaatttttgttctaatacggcaccaaagccgaatgtc |
| 17 | senseScHch1BamHI | gagagaggatccatggttgtcttgaatccaaataactg |
| 20 | antisenseScHch1mycXhoI | gagagactcgagtcactacaaatcttcttcagaaatcaatttttgttcaacttgtatatcctttgagtg |
| 27 | CScHch1BamHI | gagagaggatcctcactaaacttgtatatcctttgagtg |
| 29 | CScAha1 BamHI | gagagaggatcctcactataatacggcaccaaagccg |
| 90 | antisenseHch1mycSacI | gagagagagctctcactacaaatcttcttcagaaatcaatttttgttccataacttgtatatcctttgagtgttc |
| 126 | antisensemycAHA1NotI | gagagagcggccgctcactacaaatcttcttcagaaatcaatttttgttccattaatacggcaccaaagccg |
| 571 | senseBamHIHch1del11 | gagagaggatccatgGTGGATAAAAACACCTTACCTTGGTC |
| 572 | senseNdeIAha1del11 | gagagacatatgGTCGATAAGAACTGCATC |
| 573 | senseBamHIAha1del11 | gagagaggatccatgGTCGATAAGAACTGCATC |
| 601 | senseNdeIHch1del11 | gagagacatatgGTGGATAAAAACACCTTACCTTGGTC |
| 648 | senseBamHIHisAha1del11 | gagagaggatccatgggccatcaccatcaccatcacatgGTCGATAAGAACTGCATC |
| 649 | senseBamHIHisAha1 | gagagaggatccatgggccatcaccatcaccatcacatggtcgtgaataacccaaataactg |
| 654 | SenseNco1Hch1 | gagagaccatgGTTGTCTTGAATCCAAATAACTGGCATTG |
| 655 | SenseNco1Del11Hch1 | gagagaccatgGTGGATAAAAACACCTTACCTTGGTCTAAG |
| 656 | AntisenseBamH1CtermHisTagHch1 | gagagaggatcctcactaATGATGATGATGATGATGaacttgtatatcctttgagtg |
| 657 | SenseXba1Aha1 | gagagaTCTAGAaataatttttgtttaactttaagaaggagatataccATGgtcgtgaataacccaaataactggcactgggtcg |
| 658 | SenseXba1Del11Aha1 | gagagaTCTAGAaataatttttgtttaactttaagaaggagatataccATGgtcgataagaactgcatcggatgggccaaggag |
| 659 | AntisenseBamH1CtermHisTagAha1 | gagagaggatcctcactaATGATGATGATGATGATGtaatacggcaccaaagccgaatgtc |
| 664 | aScAha1NtermBamHICtermHistag | GAGAGAGGATCCTCACTAATGATGATGATGATGATGcacctgaatgtcattac |
| 672 | sQCHsp82S25PEcoRVremoval | caacaccgtctatcctaacaaggaaattttcttgagagaactgatctctaatgcc |
| 673 | aQCHsp82S25PEcoRVremoval | ggcattagagatcagttctctcaagaaaatttccttgttaggatagacggtgttg |

**Supplementary Figure 2.** List of primers used in this study.
